# The effect of biologically active compounds in the mucus of slugs *Limax maximus* and *Arion rufus* on human skin cells

**DOI:** 10.1038/s41598-021-98183-6

**Published:** 2021-09-21

**Authors:** Anna Leśków, Małgorzata Tarnowska, Izabela Szczuka, Dorota Diakowska

**Affiliations:** 1 Department of Nervous System Diseases, Wroclaw Medical University, 51-618 Wrocław, Poland; 2 Department of Biochemistry and Immunochemistry, Wroclaw Medical University, 50-368 Wrocław, Poland

**Keywords:** Biochemistry, Cancer, Cell biology, Drug discovery, Medical research

## Abstract

Molluscs are one of the sources of biologically active substances, which are now intensively studied, especially for their anti-cancer properties. Malignant melanoma originates from melanocytes, develops very quickly and is associated with poor prognosis. Therefore, the aim of the study was to assess the properties of biologically active compounds in sterile mucus isolated from slugs *Limax maximus* and *Arion rufus*. Tested mucus were isolated using the new self-developed method which is safe for the environment and the animal donors. The impact of the mucus on human keratinocytes CCD 1106 KERTr and malignant melanoma cells A-375 was examined using MTT assay and SRB assay, which allowed us to determine the cell metabolic activity and cell number after treating them with slug mucus isolated from *Limax maximus* and *Arion rufus* decreased human keratinocytes and melanoma cells metabolic activity as well as manifested properties of reducing the number of cells in both tested cell lines, and therefore can be a source of biologically active substances with anticancer potential.

## Introduction

Human skin, as an organ most exposed to contact with the external environment, is subjected to various damaging factors. These factors can cause pathophysiological and morphological changes or mutagenesis of the genetic material, often leading to carcinogenesis. The most common non-melanoma skin cancer (NMSC) are basal cell carcinoma (BCC) and squamous cell carcinoma (SCC), which represent 95% of all skin cancers and up to 35% of primary human malignant neoplasms^1–3^. However, the most dangerous skin cancer is malignant melanoma, which causes 1.5–2% of all malignant neoplasms, and its primary location is skin in 95% of cases^1,3,4^. Skin melanoma is a cancer with a rapid development rate and high drug resistance^3–5^. Melanoma spreads quickly to various distant organs, which makes treatment difficult or impossible. The rate of metastasis, its location and number of metastases significantly affects the survival of patients, which is why it is so important to find a way to slow this process^6,7^. Therefore, it is extremely important to implement rapid diagnosis and treatment in the early stages of melanoma, when the chances of cure are as high as 80%^3^. Currently, a cream containing imiquimod is used to treat stage 0 melanoma (melanoma limited to the epidermis)^8–10^. Imiquimod is the only topical use drug approved by the Food and Drug Administration (FDA), although its main applications are treatment of keratinization lesions, basal cell carcinoma, genital and anal warts. Based on the available data, there is currently no specific drug approved for the treatment of superficial malignant melanoma. This generates the need to search for new biologically active compounds with anti-tumor potential against this type of cancer.

One of the current trends in world science is the search for biologically active substances derived from living organisms and their use in biotechnological processes in the pharmaceutical, cosmetic, food and other industries. Numerous scientific reports indicate the cytotoxic properties of snail mucus in relation to various cell lines, including cancerous^11–19^. It was reported that *Helix aspersa maxima* snail mucus shows antitumor activity against human melanoma cells^12^ and *Cornu aspersum* snail lyophilisate significantly reduces the survival rate of Caco-2 colon cancer cells^20^. In addition, haemocyanins obtained from aquatic snails (*Rapana venosa*) and terrestrial snails (*Cornu aspersum* and *Helix lusitanicus*) are immunostimulators with antimicrobial and anticancer properties, cytotoxic to T-24 bladder cancer cells^16^. The haemolymph of aquatic *Rapana thomasina* and terrestrial *Helix pomatia* snails show antitumor activity against C-26 colorectal cancer cells^13^, and terrestrial snails *Achatina fulica* mucus is cytotoxic to MCF-7 breast cancer cells and Vero fibroblast-like renal epithelial cells^21^.

Among free-living snails in European countries, the main interest of scientists are snails *Cornu aspersum* (until 2014, known as *Helix aspersa*)^11,20,22,23^. Despite their presence in culture and natural medicine^24^, slugs did not arouse much interest among scientists, with the exception of *Limax* slugs which were behaviorally tested^25–29^.

The aim of the study was to assess the impact of the mucus of slugs *Limax maximus* and *Arion rufus* on human keratinocytes and melanoma cell lines.

## Results

### Isolation and purification of slug mucus

The developed own method of obtaining mucus is innovative and safe for both animals and material. Alcohol is not used as in other methods, which prevents protein denaturation in the formulation. The use of membrane filtration ensured the sterility of the formulations, which was confirmed by the Matrix Assisted Laser Desorption/Ionization Time-of-Flight Mass Spectrometry (MALDI-TOF MS; Table 1).

**Table 1:** Results of MALDI-TOF analysis of slug mucus.

Slug species	Step of determination	Medium type	Temperature [°C]	Species (best matching according to MALDI-TOF method	Identification value	Credibility
*Limax maximus*	Prior to filtration	BHI	30	*Pseudomonas fulva*	1.955	*
		BHI	30	*Acinetobacter radioresistens*	2.115	**
		BHI	30	*Pseudomonas fulva*	2.039	**
		BHI	30	*Acinetobacter radioresistens*	2.4	***
		BHI	30	*Acinetobacter radioresistens*	2.336	***
		BHI	30	*Enterobacter cancerogenus*	1.836	*
		AG	37	*Pseudomonas fulva*	2	**
		AG	37	*Leclercia adecaroxylata*	1.94	*
		AG	37	*Acinetobacter radioresistens*	2.341	***
		AG	37	*Pseudomonas fulva*	1.904	*
		AG	37	*Escherichia vulneris*	1.87	*
		AG	37	*Acinetobacter radioresistens*	2.293	**
		AG	37	*Escherichia vulneris*	1.952	*
	After filtration	Lack of isolates				
*Arion rufus*	Prior to filtration	AG	30	*Sphingomonas paucimobilis*	2.184	**
	After filtration	Lack of isolates				

*BHI* brain heart infusion, *AG* Agar medium.

***Reliable identification at the species level, **Reliable identification at the genus level, *Probable identification at the genus level.

### MTT assay assessment of the survival of human keratinocytes and melanomas exposed to slug mucus

**Figure 1:**
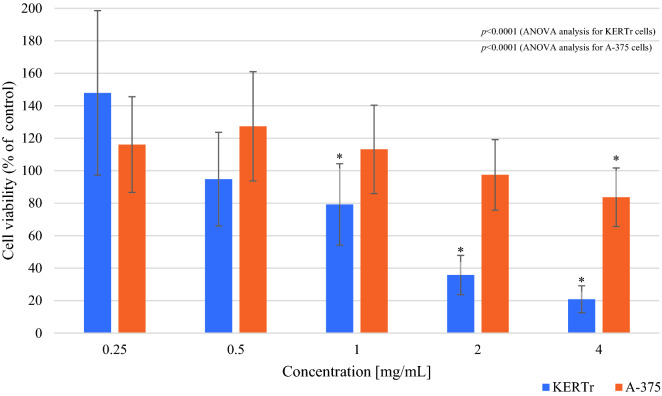
Viability of keratinocytes (KERTr) and melanoma cells (A-375) after 24 h incubation with *Limax maximus* mucus (MTT assay). Results are shown as a percentage value of living cells (control was taken as 100%). *p < 0.05 vs control.

**Figure 2:**
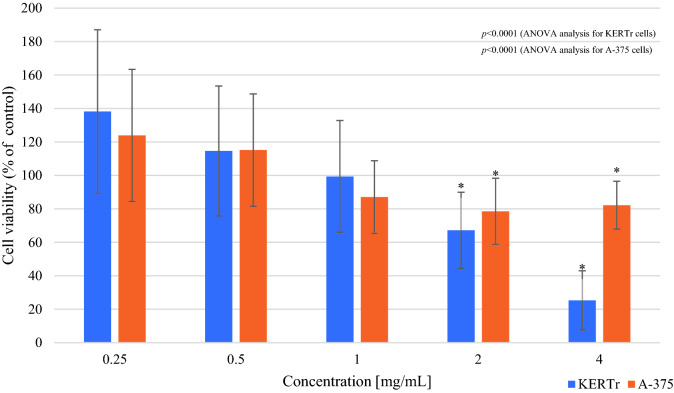
Viability of keratinocytes (KERTr) and melanoma cells (A-375) after 24 h incubation with Arion rufus mucus (MTT assay). Results are shown as a percentage value of living cells (control was taken as 100%). *p < 0.05 vs control.

The obtained data indicate a statistically significant reduction in the survival of keratinocytes in the presence of *Limax maximus* mucus at 1, 2 and 4 mg/mL concentration. The results indicate that a 4 mg/mL concentration of mucus decreased the survival of keratinocytes by 80% (cell viability 20.88 ± 8.25%; *p* < 0.0001) compared to the untreated control cells, while 2 mg/mL concentration of mucus inhibited the survival of cells by 65% (cell viability 35.78 ± 12.08%; *p* < 0.0001) (Fig. 1). In the case of *Arion rufus* mucus, the obtained data indicate a statistically significant reduction in cell survival in the presence of mucus at 2 and 4 mg/mL concentration. The results indicated that a concentration of 4 mg/mL decreased the survival of keratinocytes by 75% (cell viability 25.29 ± 17.72%; *p* < 0.0001) compared to the control, and a concentration of 2 mg/mL inhibited the survival by 35% (cell viability 67.2 ± 22.74%; *p* < 0.001) compared to control (Fig. 2).

In the case of melanoma, one-way ANOVA analysis showed statistically significant differences in cell viability between tested groups incubated with mucus of *Limax maximus* or *Arion rufus* (*p* < 0.0001) (Figs. 1, 2). There was a significant reduction in the survival of melanoma cells by 17% (cell viability 83.67 ± 18.02%; p = 0.034) when exposed to *Limax maximus* mucus at 4 mg/mL concentration (Fig. 1). In the case of *Arion rufus*, the results show a significant reduction in the survival of melanoma cells treated with mucus at 2 and 4 mg/mL, reducing the number of viable cells by 22% (cell viability 78.53 ± 19.78%; *p* = 0.006) and 18% (cell viability 82.22 ± 14.3%; *p* = 0.035), respectively (Fig. 2). The results showed that 2 mg/mL *Arion rufus* mucus caused the greatest change in melanoma cell survival, leading to a 22% decrease in viable cell number compared to untreated control cells.

**Figure 3:**
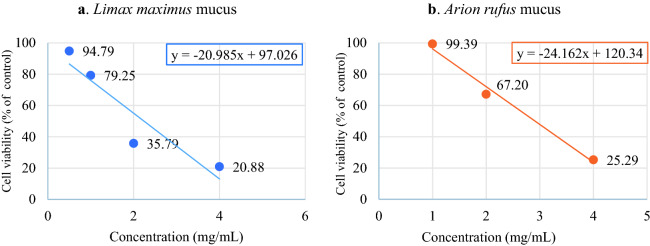
Half maximal inhibitory concentration (IC_50_) calculated for *Limax maximus* mucus (**a**) and *Arion rufus* mucus (**b**) based on the MTT assay results.

On the basis of the obtained data, the value of the minimum inhibitory concentration was calculated, defined as the minimum concentration of the tested compound needed to inhibit the growth of the tested cells by 50% (IC_50_), which in the case of *Limax maximus* slug mucus was IC_50_ = 2.35 mg/mL, and for *Arion rufus* mucus IC_50_ = 2.91 mg/mL (Fig. 3.).

### Sulforhodamine B assay assessment of the survival of human keratinocytes and melanomas exposed to slug mucus

In the sulforhodamine B (SRB) assay, 1, 2 and 4 mg/mL *Limax maximus* mucus showed statistically significant cytotoxic effects which caused, compared to the cell number in control group, a decrease in the number of keratinocytes by 14% (cell number 86.23 ± 7.67%; *p* < 0.0001), 25% (cell number 75.22 ± 4.36%; *p* < 0.0001) and 18% (cell number 82.12 ± 4.84%; *p* < 0.0001), respectively. The obtained results for the concentrations of 2 mg/mL and 4 mg/mL of *Arion rufus* mucus indicated a statistically significant cytotoxic effect of these mucus towards keratinocytes, reducing their number by 19% (cell number 81.51 ± 5.73%; *p* = 0.025) and 35% (cell number 66.85 ± 3.9%; *p* < 0.0001) respectively. The most cytotoxic to keratinocytes were *Limax maximus* mucus at a concentration of 2 mg/mL and 4 mg/mL *Arion rufus* mucus, causing a decrease in the number of cells by 25% and 35%, respectively.

**Figure 4:**
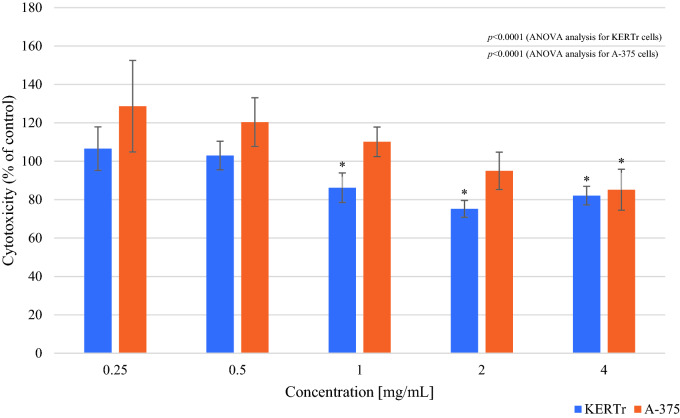
The survival of keratinocytes (KERTr) and melanoma cells (A-375) after 24 h incubation with *Limax maximus’* mucus (SRB assay). Results are shown as a percentage value of living cells (control was taken as 100%). *p < 0.05 vs control.

**Figure 5:**
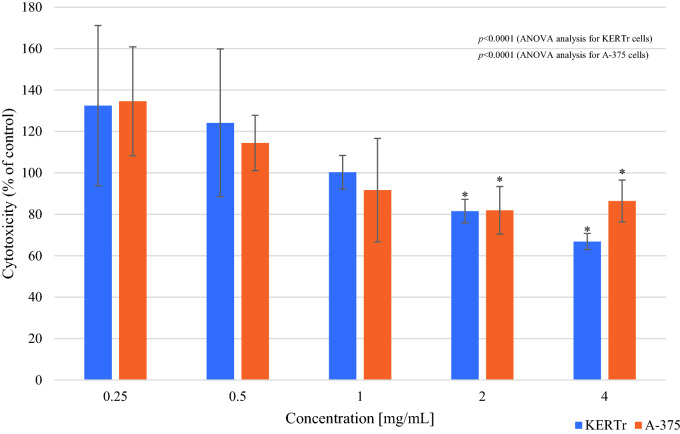
The survival of keratinocytes (KERTr) and melanoma cells (A-375) after 24 h incubation with *Arion rufus’* mucus (SRB assay). Results are shown as a percentage value of living cells (control was taken as 100%). *p < 0.05 vs control.

In the case of A-375 melanoma cells, statistically significant differences in cell viability were observed between tested groups after incubation with *Limax maximus* or *Arion rufus* mucus (*p* < 0.0001 for both) (Figs. 4, 5). 4 mg/mL *Limax maximus* mucus reduced cell number by 15% (cell number 85.17 ± 10.65%; *p* < 0.001). The most cytotoxic mucus for A-375 melanoma cells was *Arion rufus* mucus at a concentration of 2 mg/mL, which reduced cell survival by 18% (cell number 81.93 ± 11.49%; *p* = 0.001) (Fig. 5).

## Discussion

Slugs naturally occurring in Western Europe, *Limax maximus* and *Arion rufus*, have not yet been tested for their biological properties in relation to human keratinocytes and melanoma cells. Existing research papers on proteolytic^30^, anti-bacterial^22,31,32^, anti-cancer^11,12,20^ properties prompted an in-depth analysis of the biological properties of these slugs' mucus. For the research described in this study, slug mucus was obtained by own method, non-invasive for slugs, using low (0.9%) sodium chloride concentration. The obtained mucus, in a freeze-dried form, is sterile and could be safely stored for further analysis. Many methods of obtaining mucus from slugs and snails were described, including manual stimulation^12,22,31,33^, electrical stimulation^20^ and even by cutting the bodies of slugs/snails and squeezing out the mucus^34^, or whole animal homogenization^32^. Swapna et al. even describes obtaining mucus through ethanol extraction^33^. The main limitation in the case of this study was the small amount of mucus produced by slugs, which prevents efficient mucus collection using manual stimulation. Incision or other damage to the body of slugs/snails, including homogenization of whole animals, are extreme and violent, and methods based on high salt concentrations may impede further research and even cause degradation and formation of non-specific particles in the tested material^23,35^. The own method of isolation, used in the above-described studies, allows to isolate clean mucus, without contamination with cells or animal material (e.g. haemolymph). Additionally, based on our other studies, we did not notice any change in the composition of the mucus, regardless of the year of the slugs harvest. Therefore we conclude, obtained mucus has a constant composition, although in general external and environmental factors may have an influence on slugs and their mucus (data not published).

The results obtained in the process of our own research showed that the tested mucus of the slugs *Limax maximus* and *Arion rufus* differ from each other in terms of their effect on human keratinocytes and melanoma cells, both in terms of their impact on the survival of these cells and the nature of this effect, i.e. cytotoxic properties. The use of two different assays: MTT and SRB, made us possible to show the differences in mucus activity. MTT measures cell metabolic activity, which depends on cell viability but also on cell number. SRB measures cell total protein, which depends on a cell cycle phase but more directly gives an indication about the cell number. The results of the MTT and SRB assays demonstrated the same tendency: the influence of slug mucus on keratinocyte culture is cytotoxic, and the number of keratinocyte cells decreases with increasing mucus concentration. When comparing the results obtained with the MTT assay with the results of the SRB assay, the concentration of 2 mg/mL resulted in a strong decrease of cell metabolic activity and viability, but the total protein mass was less affected. Taking into account the principles of the MTT and SRB assays and on the basis of the presented data, it can be concluded that *Limax maximus* mucus does not inhibit cell growth in the initial phase of cell culture. Comparing the results and observations to the data obtained in the study of *Limax maximus* mucus, in the case of *Arion rufus* mucus, the principle of action is analogous. However, additional tests, with more timepoints, are needed to see if this effect may be based on a delayed cytotoxic effect against keratinocyte cells in the late growth phase. It is also worth noting that due to the presence of sugars in the mucus of slugs, it has a thick consistency, which in high concentrations may negatively affect the bioavailability of nutrients contained in the culture medium, which may additionally determine the higher cell death at high concentrations of the tested mucus.

Therefore, *Limax maximus* slug mucus shows a stronger cytotoxic effect against human keratinocytes, which determines its influence on the decrease in the survival of these cells, than in the case of *Arion rufus* mucus. In turn, melanoma cells are more sensitive to the action of *Arion rufus* mucus, showing higher mortality in the environment containing this mucus.

Due to the lack of scientific reports describing the influence of *Limax maximus* and *Arion rufus* slug mucus on the survival of human cells, the results of our research were compared to studies on the mucus of other slugs and snails and/or other mixtures obtained from snails/slugs of various species. Matusiewicz et al.^20^ investigated the effect of *Cornu aspersum* snail mucus, homogenate from the body of this snail and snail shells dust, indicated that after 24 h of incubation of Caco-2 colon cancer cells with snail mucus at a concentration of 2.5 mg/mL, cell survival measured by the MTT assay was higher than the values obtained for untreated control cells. On the other hand, 72 h incubation caused a decrease in cell survival by about 25%. Studies on the effect of *Achatina fulica* snail mucus on MCF-7 breast cancer cells have shown that this mucus at a concentration of 1 mg/mL after 72 h of incubation reduced the survival rate of these cells by 20%^21^. In addition, the studies of other authors have shown that haemocyanins obtained from the hemolymph of the water snail *Rapana venosa* and two terrestrial snails: *Cornu aspersum* and *Helix lucorum*, exhibit anti-cancer properties against bladder cancer^11,15,16,36^. These properties, however, were strongly dependent on the incubation time, concentration of the tested compounds and their quality, i.e. the type of the tested haemocyanin subunit^11,16^.

In conclusion, the subject of own research is adding more data to the field of the properties of biologically active substances obtained from slugs and points to the need to continue research and expand knowledge in this field.

On the basis of the obtained results, it was found that the mucus of slugs *Limax maximus* and *Arion rufus* differ in terms of biological properties in relation to human cells (normal keratinocytes and melanoma cells). The mucus of slugs has strong properties that reduced the survival of keratinocytes and reduced the number of melanoma cells by up to 22%. *Limax maximus* mucus showed a stronger influence on cell survival, and *Arion rufus* mucus was characterized by higher cytotoxicity in relation to both tested cell lines. However, further research is needed to determine the mechanisms of action of the mucus.

## Methods

### Animal material and preparation of samples

The material used in the study was the mucus of slugs: *Limax maximu*s (commonly named as leopard slug or great grey slug) and *Arion rufus* (known as European red slug).

*Limax maximus* and *Arion rufus* slugs have been harvested from a garden, located in a green district of the city, bordering a park and other wild gardens, without contact with city streets. No plant protection products nor fertilizer (and other similar products) were used in this area as well as in the closest neighborhood. We collected only adult individuals, based on the criterium of body length—more than 10 cm for *Limax maximus* and 8 cm in the case of *Arion rufus*^37–39^. The slugs (32 *Limax maximus* specimens and 34 *Arion rufus* specimens) were shaken with 0.9% NaCl (B. Braun, Germany) for 20 min, at 150 rpm, at room temperature. The mucus solution was then centrifuged at 10,000×*g*, 15 min, 4 °C. The supernatant was poured into dialysis tubes (Carl Roth GmbH, Germany) and desalted at 4 °C. The dialysate was filtered into a sterile bottle through 0.22 pm membrane filters (Millipore Express PLUS from PES—Sigma-Aldrich, USA) placed in a Nalgene ™ Polysulfone Reusable Bottle Top Filters (Thermo Fisher Scientific, Waltham, MA, USA). The solution was then sterile poured into sterile containers, frozen at − 80 °C and lyophilized. The lyophilisate was stored in tightly closed containers at − 20 °C for further research.

After the extraction, slugs were placed in safe, single containers located in their natural habitat and after about 2 weeks they could be used again (no more than 3 times) or were set free. A period of 2 weeks was needed for full recovery, due to the fact that after the contact with NaCl slugs were less elastic and dehydrated.

### MALDI-TOF MS analysis of slug mucus lyophilisates purity

The research material consisted of aqueous solutions of *Limax maximus* and *Arion rufus* mucus. Matrix Assisted Laser Desorption/Ionization Time-of-Flight Mass Spectrometry—MALDI-TOF MS^40–42^ technique was used to determine microbiological purity of mucus before and after filtration. The tested materials were seeded on the media: BHI ((Brain Heart Infusion)—Oxoid, Wielka Brytania), AG ((Agar medium)—BTL Sp. z o.o., Poland), BA ((blood agar = AG + bovine heart blood)—BTL Sp. z o.o., Poland), STG ((AG + sodium thioglycolate)—IITD PAN, Wroclaw, Poland), YM ((yeast extract + malt extract agar)—IITD PAN, Wroclaw, Poland), and incubated for 24 h at 30 °C and 37 °C under aerobic and anaerobic conditions. Bacteria isolated from single homogeneous colonies were identified using the MALDI-TOF MS technique. The procedure included an initial step: a bacterial colony was dissolved in 300 µL of sterile deionized water, 900 µL of ethanol (POCH, Poland) was added and thoroughly mixed. The solution was then centrifuged at 13,000×*g* for 2 min, 50 µL of a 70% aqueous formic acid solution (Pol-Aura, Poland) and 50 µL of acetonitrile (Pol-Aura, Poland) were added to the pellet and thoroughly mixed, followed by centrifugation. The results were compiled on the basis of the MALDI-Biotyper 3.0 database (Bruker, Germany) according to the point scale: 3.000–2.300 reliable identification of the microorganism at the species level; 2.299–2.000 reliable identification of the microorganism at the genus level and highly probable at the species level; 1.999–1.700 probable result at genus level; 1.699–0 no reliable identification.

### Determination of the cytotoxic activity of slug mucus on selected human cell lines

Human keratinocytes CCD 1106 KERTr (ATCC® CRL2309 ™) were obtained from the American Type Culture Collection (ATCC; MD, USA) and were cultured in a medium composed of Keratinocyte Serum Free Medium (Gibco, Thermo Fisher Scientific, Waltham, MA, USA) and dedicated supplements: 30 µg/mL bovine pituitary extract (BPE; Gibco) and 0.2 ng/mL recombinant human endothelial growth factor (rEGF; Gibco) with addition of stabilized 1% antibiotic antimycotic solution containing 25 µg/mL of amphotericin B, 10,000 units of penicillin/mL, 10 mg/mL of streptomycin (Sigma-Aldrich, St. Luis, MO, USA). For the experiments CCD 1106 KERTr cells were seeded at 4 × 10^3^ cells per well on a 96-well plate and cultured for 24 h in CELCULTURE^®^ CCL-170B-8 CO_2_ incubator (Esco, Singapore) at 37 °C in 95% air with 5% CO_2_.

Human malignant melanoma A-375 cells (ATCC; ATCC^®^ CRL1619™) were cultured in Dulbecco's Modified Eagle’s Medium (DMEM; Gibco) supplemented with 10% fetal bovine serum (FBS; Sigma-Aldrich, USA) with addition of stabilized 1% antibiotic antimycotic solution (Sigma-Aldrich). For the experiments A-375 cells were seeded at 2 × 10^3^ cells per well on a 96-well cell culture treated plate and cultured for 24 h in the CO_2_ incubator at the above-mentioned conditions.

Cells from both cell lines were treated for 24 h with *Arion rufus* or *Limax maximus* mucus dissolved in dedicated complete growth medium at 0.25, 0.5, 1, 2, and 4 mg/mL concentration. Cells cultured with dedicated complete growth medium alone were used as controls. Mucus were added to the same number of cells both in MTT and SRB assays, and also in the same culture stage.

Both assays were conducted at 1 day, from one cell culture. The experiments were done in a triplicate.

### Evaluation of cell metabolic activity of cells treated with slug mucus with the MTT assay

After 24 h of treatment, post-culture medium was discarded, cells were rinsed with sterile phosphate-buffered saline (PBS) solution and 0.5 mg/mL 3-(4,5-dimethylthiazol-2-yl)-2,5-diphenyl tetrazolium bromide in complete growth medium (MTT reagent; Sigma-Aldrich) was added. Plates were incubated for 3 h in the CO_2_ incubator at the above-mentioned conditions. Subsequently, the MTT reagent was decanted and the formed formazan crystals were dissolved by dimethyl sulfoxide (DMSO; BioShop, Canada). The absorbance was measured using an Infinite^®^ M200 plate spectrophotometer (Tecan Group Ltd., Männedorf, Switzerland) at λ = 540 nm.

### Evaluation of the cells number after exposure to slug mucus with the sulforhodamine B (SRB) assay

After 24 h of treatment, cells were fixed with 12.5% trichloroacetic acid (TCA; Sigma-Aldrich) and incubated for 1 h at 4 °C, followed by rinsing with distilled water and drying. Subsequently, a freshly prepared solution of 0.04% SRB (Sigma-Aldrich, USA) in 1% acetic acid (Avantor Performance Materials Poland, Poland) was added and let stand for 30 min. The unbound dye was removed using 1% acetic acid. The protein-bound SRB was solubilized by 10 mM Tris base solution (pH 10.5). The absorbance, proportional to the protein content, was measured using an Infinite® M200 plate spectrophotometer (Tecan Group Ltd) at λ = 520 nm.

### Statistical analysis

Statistical analysis was conducted using MS Excel 2016 (Microsoft Co, USA) and Statistica 13.3 (Tibco Software Inc., CA, USA). Descriptive data were presented as a mean and a standard deviation. Distribution of the data was tested with the Shapiro–Wilk normality test, and homogeneity of variances were analyzed by the Leven’s test. One-way ANOVA analysis and post-hoc LSD test were performed for evaluation of differences between independent groups. In all analyzes a 2-tailed p-value of *p* < 0.05 was considered statistically significant.

## Conclusions

1. The developed own method of isolating and purifying the mucus of the *Limax maximus* and *Arion rufus* slugs ensures the sterility of the obtained product, which is a condition for working with it in safe conditions. Thanks to the final, freeze-dried form of the product, it is also possible to store and easily dissolve it in various solvents, which determines a wide range of applications.
2. *Limax maximus* and *Arion rufus* slug mucus significantly reduce the survival of human keratinocytes and malignant melanoma cells.
3. *Limax maximus* and *Arion rufus* slug mucus are cytotoxic to both human keratinocytes and malignant melanoma cells. Further research is required to determine the mechanisms of the toxic effect underlying the decreased cell survival.

## Data Availability

All data are available within the text of the article. The raw/processed data required to reproduce these findings are available from the corresponding author upon reasonable request.
