# Author Correction: The effect of biologically active compounds in the mucus of slugs *Limax maximus* and *Arion rufus* on human skin cells

**DOI:** 10.1038/s41598-021-99324-7

**Published:** 2021-09-27

**Authors:** Anna Leśków, Małgorzata Tarnowska, Izabela Szczuka, Dorota Diakowska

**Affiliations:** 1 Department of Nervous System Diseases, Wroclaw Medical University, 51‑618 Wrocław, Poland; 2 Department of Biochemistry and Immunochemistry, Wroclaw Medical University, 50‑368 Wrocław, Poland

Correction to: *Scientific Reports* 10.1038/s41598-021-98183-6, published online 21 September 2021

The original version of this Article contained errors in the spelling of the authors Anna Leśków, Małgorzata Tarnowska, Izabela Szczuka & Dorota Diakowska which were incorrectly given as Leśków Anna, Tarnowska Małgorzata, Szczuka Izabela & Diakowska Dorota respectively.

The original Article has been corrected.

